# Measuring the Relative Utility Loss of Legitimacy Deviation: A Discussion Based on the Public Goods Experiment

**DOI:** 10.3390/bs13050366

**Published:** 2023-04-28

**Authors:** Wenjie Zhang, Xianchen Zhu, Hongyu Guan, Tao Li

**Affiliations:** 1 School of Economics and Management, Nanjing University of Science and Technology, Nanjing 210094, China; 2 Center for Experimental Economics in Education, Shaanxi Normal University, Xi’an 710119, China

**Keywords:** legitimate behavior, deviation, measurement, conditional cooperation, utility function

## Abstract

In order to understand the differences in individual behavior across different contexts, this study introduces legitimate behavior and its deviation into a utility function. We hypothesize that people have preferences for adhering to the legitimate behavior that is required by the behavioral norm embedded in a particular context; furthermore, deviating from this legitimate behavior may generate a utility loss for them. We apply our model in the context of conditional contributions in a public goods experiment; moreover, we verify that the behavioral pattern of this conditional cooperation is derived from subjects’ preferences for complying with the legitimate behavior required by the norm of the conditional cooperation activated in the experimental context. Furthermore, we attempt to measure the individual-level degrees of respect for the legitimate behavior in the given context using observable experimental data. The measurement results reveal that the subjects’ relative sensitivities to deviations are highly centrally distributed; additionally, most subjects have a relatively high degree of respect for the legitimate behavior required by the conditional cooperation norm. Accordingly, this paper will help to improve our understanding of the micro mechanism underlying individual behavior.

## 1. Introduction

Based on the standard rational choice theory with selfish preferences, the optimal choice of rational participants in a standard one-shot public goods experiment contributes nothing, i.e., free-riding. However, numerous experimental results have shown that non-zero contributions prevail and people cooperate at considerably higher levels than theoretically expected. The common explanation for this is that individuals are likely to follow the conditional cooperation norm [1] or the non-outcome-oriented norm of fairness [2]: they will cooperate if and only if other people cooperate. Specifically, in public goods experiments, people who follow the conditional cooperation norm, i.e., conditional cooperators, make decisions depending on others’ actions or their expectations about how others will act [3,4]; their contributions increase as their expectations about the level of others’ cooperation rise. Thus, a non-zero contribution to public goods is possible for a heterogeneous preference group containing both self-interested participants and conditional cooperators.

To verify the existence of conditional cooperation, Fischbacher et al. [5] introduced the “strategy method” proposed by Selten into a public goods experiment to identify the subjects’ heterogeneous types of preference. Specifically, the subjects were requested to decide how much they were willing to contribute to the public good given the possible average contribution levels of the other group members. Their experimental results demonstrated that most subjects’ contribution behavior displayed conditional cooperation: their contribution increased with the other group members’ average contribution level. Moreover, the majority of the conditional cooperators were partially conditionally cooperative since they generally contributed less than the others’ average contribution. These findings indicate that subjects may also consider monetary payoffs when following the conditional cooperation norm, which weakens the extent of their adherence to the norm. Numerous laboratory and field experiments have confirmed these results [4,6,7].

In the current literature, issues concerning the identification of the behavioral pattern of conditional cooperation have been well investigated. However, researchers have paid insufficient attention to explaining the micro mechanism underlying this conditional cooperation behavior through simple models. Fehr and Schmidt [8] assumed that people are motivated by fairness considerations and formalized the notion of fairness by introducing the preference of inequity aversion into the utility function; inspired by them, we attempted to interpret individuals’ behavioral patterns in given contexts from the micro-level with a single general model and to formalize the preference of conditional cooperation through this.

Previous research based on the social preference theory has constructed utility functions mainly by assuming that people care about others’ material payoffs and make a trade-off between their own material payoffs and others’ when making a choice [8,9,10,11]. However, many empirical results still cannot be explained by such utility theories, which are based solely on individuals’ preferences for the relative distribution of material benefits. For example, List [12] found that, in the dictator experiment, extending the choice set of dictators by offering the opportunity to take money from the recipient may influence subjects’ decisions substantially. Falk et al. [13] declared that the rejection rates of responders in the ultimatum game depended on the choice set available to the proposers. Moreover, many studies have found that behavioral experiments with the same payoff structure, however, differ in their experimental background descriptions and may have significantly different results [14,15], indicating that individuals’ behavior is sensitive to contexts. In the field of psychology, Tversky and Kahneman [16] defined this phenomenon as the framing effect. They also proposed the prospect theory [17], which emphasizes the effect of contexts and references on people’s preferences and choices. Evidently, these phenomena are inconsistent with outcome-based distributional preferences.

To explain this puzzling evidence, we suppose that people have preferences for following the behavioral norm embedded in a given context. The norm implies what the right thing to do is, which we call the legitimate behavior, in the given context. That is, individuals have preferences for complying with this legitimate behavior beyond the material payoffs induced by the behavior, and they do so in a context-sensitive manner. Specifically, we introduce behavior legitimacy into utility function and assume that context signals will activate individuals’ normative perception about the legitimate behavior required by the norm, which is context-dependent [18]. People who have a preference for adhering to the legitimate behavior will have unpleasant emotions such as guilt or remorse and suffer utility loss if their action deviates from the behavioral standard of legitimacy. Additionally, individuals’ sensitivity to deviations, which depends on the extent of their adherence to the legitimacy, is heterogeneous. People will trade-off between their material payoffs and the utility loss that they may experience if they deviate from the legitimate behavior when making a decision. According to our model, the above-mentioned perplexing evidence can be explained as the heterogeneity in the legitimate behavior or behavioral norm activated by different context signals.

Based on the decision model, we subsequently formalize the preference of conditional cooperation and explain the micro mechanism underlying it. Specifically, we argue that, in the decision situation designed in Fischbacher et al.’s [5] conditional contribution of the public goods experiment, subjects who had internalized the norm of conditional cooperation regarded the other group members’ average contribution as the standard behavior with a legitimacy that they should choose. Furthermore, they chose their contribution by making a trade-off between the utility improvement and loss from their monetary payoffs and deviations from the legitimate behavior, respectively. The analytical results of our utility function demonstrate that: purely self-interested subjects are insensitive to deviations from the legitimate behavior and only care about their own monetary payoff, and regardless of how much the others contribute to the public good, their optimal choice contributes nothing; however, for conditional cooperators, deviating from the legitimate behavior will cause an immaterial utility loss, and their optimal choice is a linear function of the others’ average contribution, which, in turn, confirms that the behavioral pattern of conditional cooperation can be explained by this paper’s analytical framework. We apply our general model to the context of conditional contribution in the public goods experiment to explain the behavioral pattern of conditional cooperation in this paper. Meanwhile, the formalization of the concept of legitimacy deviation can also explain other moral behavior such as promise-keeping and honesty [19], which is worthy of further investigation.

In addition, according to the equilibrium analysis of the utility function, we obtain an equation that represents the degree of an individual’s adherence to the legitimate behavior relative to his or her preference for monetary payoffs (which will be discussed in the rest of this paper). In order to provide a more detailed picture of the distribution of individuals’ heterogeneous degree of adherence to the legitimate behavior, we conducted an experiment similar to the conditional contribution experiment by Fischbacher et al. [5] and utilized the observable conditional contribution data to measure the parameter representing each subject’s relative degree of adherence [20,21]. The measurement results indicate that the subjects’ relative sensitivities to deviations are highly centrally distributed; furthermore, most subjects have a relatively high degree of adherence to the legitimate behavior in the experimental context of conditional contribution. Using our model, the measurement of each subject’s degree of compliance with the conditional cooperation norm helps to interpret the subjects’ choices in a more general way, compared to the identification of the behavioral pattern of conditional cooperation.

The rest of this paper is organized as follows. Section 2 reviews the related literature. Section 3 presents the model containing the legitimate behavior and its deviation, as well as describes the experimental details and procedures. Section 4 presents the experimental results and analyses. Section 5 concludes with some discussion.

## 2. Literature Review

Numerous experimental evidence has suggested that people care about others’ benefits and exhibit considerably more prosociality than predicted in the standard self-interest model. Previous studies have attempted to interpret such observations by assuming that people have other-regarding preferences and modifying individuals’ utility function based on their preferences over the relative distribution of material payoffs. For example, Edgeworth [9] first proposed a model of altruism where individuals’ utility was a weighted sum of their own and others’ material payoffs. Rabin [10] took people’s intentions into consideration and pioneered a model of reciprocity [11,22,23]. According to Rabin [10], people want to be kind (unkind) to those whose intentions are kind (hostile); the kindness or the hostility of others’ intentions depends on the equity of the payoff distribution caused by their actions. Therefore, Rabin’s reciprocity model was still based on distributional preferences. Moreover, Fehr and Schmidt [8] modelled people’s inequity aversion by supposing that they resist inequitable outcomes and introduced relative material payoffs into the utility function [24].

Despite the above-mentioned research regarding the formalization of social preferences, there remains ample experimental evidence that cannot be accounted for, which we have discussed in the previous section. These data suggest that people’s behavior seems to be sensitive to contexts, and utility theories based only on distributional preferences cannot explain this phenomenon. Nevertheless, some scholars have pointed out that individuals have an evaluation of their behavior that is independent of the distribution of material payoffs caused by the behavior, such as “warm-glow” [25]. In the Theory of Moral Sentiments, Adam Smith [26] proposed that people decide whether to follow the rules of conduct according to propriety. Smith also emphasized that distinguishing the utility of the behavioral outcome from that of the behavior itself could help with understanding why decision contexts influence individuals’ choices. Therefore, considerable attention should be paid to people’s preferences about behavior, per se [27]. Along this line, researchers have adopted a new approach by assuming that people have a desire to adhere to social norms [14,20,21,28,29,30,31]; social norms are defined as the standards of behavior that are based on widely shared beliefs about how individual group members ought to behave in a given situation. In their opinion, prosocial behavior is not directly driven by preferences for material payoff distributions, rather by preferences for complying with social norms. According to their models, people evaluate the appropriateness of their behavior by comparing it to social norms and may suffer disutility from violating these norms. Furthermore, Bicchieri and Chavez [32] proposed that people have preferences for following personal norms, which are defined as internal standards about what the right thing is to do in a given context, beyond the material payoffs caused by the behavior in one-shot anonymous interactions.

Analogous to their idea, we argue that people have preferences for complying with the legitimate behavior in a specific context; this emphasizes the conception that people have preferences about their behavior and not only about the distribution of material payoffs induced by this behavior. We assume that people internalize behavioral norms by observing or experiencing punishments for violations of these norms or rewards for adherence to them; this shapes their preferences for a specific behavior in a given situation. Furthermore, when they enter a decision situation, their knowledge regarding the most appropriate action that they should take, which is implied by the behavioral norm embedded in the context, is activated (We call the appropriate action the legitimate behavior. The idea of legitimacy has been well investigated in psychology and law. In response to the issue “why people obey the law”, Tyler [33] proposed that people’s respect for legitimacy, rather than the avoidance of punishments, is the main reason for obeying the law. Andrighetto et al. [34] proposed that perceived legitimacy can motivate people to comply with norms. In the area of economics, similar to Tyler’s opinion, Fehr and Fischbacher [1] pointed out that legal enforcement mechanisms cannot function unless they are based on a broad consensus about the normative legitimacy of the rules). Deviating from the standard of legitimate behavior triggers negative emotions such as guilt or remorse and causes utility loss (If individuals’ behavior is observable to others, deviations from the legitimate behavior may also cause unpleasant emotions such as shame or embarrassment). People may adjust their decisions to prevent the activation of these negative emotions, even when their decisions are unobservable and external sanctions are unlikely.

Subsequently, based on the formalization of the idea that people suffer a utility loss upon deviating from the legitimate behavior, we measure the parameter representing individuals’ sensitivity to such deviations, relative to their preferences for monetary payoffs, using observable experimental data; this helps to understand their choices in a more intuitive way. However, existing studies about the measurement of these preferences have concentrated on evaluating people’s sensitivity to the relative distribution of material outcomes caused by their behavior; furthermore, insufficient attention has been paid to measuring their preferences for the behavior itself. For example, Fehr and Schmidt [8] constructed a linear utility function and measured the parameter representing individuals’ sensitivity to negative feelings, such as guilt, arising from their material advantage with the experimental data. Murphy et al. [35] measured the magnitude of people’s social value orientation, which represents their motivations when choosing among the interdependent outcomes using a slider experiment. Thus, this paper attempts to fill this gap in the literature by measuring individuals’ relative preferences for adhering to the legitimate behavior using observable experimental data.

## 3. Materials and Methods

### 3.1. The Model

#### 3.1.1. The Model Containing the Legitimate Behavior and Its Deviation

According to the aforementioned analysis idea, the utility function of an individual that contains the legitimate behavior and its deviation is represented as follows:

(1) $$u_{i}=u_{i}\left(W_{i};D_{i}\right)=u_{i}\left[W\left(x_{i}\right);\left|N-x_{i}\right|\right].$$

Here, $W\left(x_{i}\right)$ is the material payoff induced by an individual *i*’s actual behavior $x_{i}$; an increase in the individual’s material payoff will generate utility improvement. *N* denotes the legitimate behavior that is required by the behavioral norm activated in a given situation. Furthermore, $D_{i}=\left|N-x_{i}\right|$ represents the extent of individual *i*’s deviation from the legitimate behavior. A negative deviation ($x_{i}<N$) may trigger unpleasant emotions such as guilt or remorse and generate utility loss, while a non-deviation ($x_{i}=N$) or positive deviation ($x_{i}>N$) may generate utility improvement. The degree of utility loss or improvement for the same deviation is heterogeneous, depending on individuals’ sensitivity to these deviations. When making decisions, individuals will trade-off between material payoffs and the utility loss that they may suffer due to deviating from the legitimate behavior. Purely selfish people only care about their own material payoff and are insensitive to such deviations, thus their optimal choice is to maximize the former.

#### 3.1.2. The Case Study of Conditional Contributions in the Public Goods Experiment

In a standard one-shot public goods experiment, *M* players anonymously participate as a group. Each player has an initial endowment of *w* and decides the amount they contribute to the group’s public account, which is denoted as $x_{i}\in \left[0,w\right]$. The total amount contributed to the group’s public account is multiplied by γ, and subsequently equally shared by the *M* group members, where 1<γ<M [36].

In the experiment designed by Fischbacher et al. [5], which was employed to verify the existence of conditional cooperation, the “strategy method” was introduced into the public goods experiment. The subjects needed to decide the amount they were willing to contribute to the public goods given the other group members’ possible average contributions, thus implying the signal of conditional contribution. In this circumstance, the experimental context signal would activate the conditional cooperation norm, which requires someone to cooperate only if others do so too, and is internalized by individuals. Therefore, those who had a preference for adhering to the norm of conditional cooperation would regard the other group members’ average contribution as the legitimate behavior they needed to demonstrate in this experimental setting.

In the public goods experiment, $x_{e}=\left(\sum x_{-i}\right)/\left(M-1\right)$ represents the average contribution of the other group members, where $\sum x_{-i}$ denotes the total amount the other group members contribute. Moreover, $x_{e}$ is the legitimate behavior that should be taken in the conditional contribution situation by the one who has internalized the norm of conditional cooperation; $D_{i}=\left|N-x_{i}\right|=\left|x_{e}-x_{i}\right|$ represents the extent of the deviation of individual *i*’s choice $x_{i}$ from the legitimate behavior $x_{e}$. According to Equation (1), the utility function of individual *i* is denoted as:

(2) $$u_{i}=u_{i}\left(W_{i};D_{i}\right)=u_{i}\left[W\left(x_{i}\right);\left|x_{e}-x_{i}\right|\right],$$

where $W\left(x_{i}\right)=w-\left(1-\gamma /M\right)x_{i}+\left(\gamma /M\right)\sum x_{-i}=w-\left(1-\gamma /M\right)x_{i}+\left(\gamma /M\right)\left(M-1\right)x_{e}$.

Specifically, we adopt the log-linear form of the utility function (2) in the following analysis and discuss the optimal choice of individual *i* in the decision situation. Subsequently, the utility function of individual *i* takes the following form:

(3) $$u_{i}=u_{i}\left(W_{i};D_{i}\right)=a_{i}\ln W_{i}+\lambda \cdot b_{i}\ln\left(c+D_{i}\right)=a_{i}\ln[w-\left(1-\gamma /M\right)x_{i}+\;\left(\gamma /M\right)\left(M-1\right)x_{e}]+\lambda \cdot b_{i}\ln\left(c+\left|x_{e}-x_{i}\right|\right)\;0\leq x_{i}\leq w.$$

Here, $a_{i}>0$ represents how intensely individual *i* cares about his or her material payoff; $b_{i}\geq 0$ indicates *i*’s sensitivity to deviations from the legitimate behavior. Considering that a non-deviation from the legitimate behavior, that is, $x_{i}=x_{e}$, may generate a positive utility for an individual, we introduce a constant *c* which is greater than 1. In order to differentiate between the utility loss caused by negative deviations and the utility improvement from a non-deviation or positive deviations, we introduce the parameter *λ*, the value of which is equal to −1 in the case of $0\leq x_{i}<x_{e}$ and 1 in the case of $x_{e}\leq x_{i}\leq w$. Furthermore, we analyze individual *i*’s optimal behavior in the conditional contribution situation by the first-order condition.

If $b_{i}=0$, individual *i* is a purely self-interested person who only cares about his or her own material payoff. Therefore, the optimal choice for individual *i* is to contribute nothing, that is, $x_{i}^{*}=0$.

If $b_{i}>0$, individual *i* has internalized the norm of conditional cooperation and is sensitive to deviations from the legitimate behavior. The discussion about the optimal choice for this type of individual has been divided into the following two cases.

The first case is when $0\leq x_{i}<x_{e}$, that is, individual *i*’s behavior negatively deviates from the legitimate behavior. Then, the utility function of *i* is written as follows:

(4) $$u_{i}=a_{i}\ln\left[w-\left(1-\gamma /M\right)x_{i}+\left(\gamma /M\right)\left(M-1\right)x_{e}\right]-b_{i}\ln\left(c+x_{e}-x_{i}\right).$$

According to the first-order condition, the optimal behavior for individual *i* is:

(5) $$x_{i}^{*}=\left(\frac{a_{i}-b_{i}\cdot \delta }{a_{i}-b_{i}}\right)\cdot x_{e}+\frac{a_{i}\cdot c}{a_{i}-b_{i}}-\frac{b_{i}\cdot w}{a_{i}-b_{i}}/\left(1-\gamma /M\right),$$

where $\delta =\left(\gamma /M\right)\left(M-1\right)/\left(1-\gamma /M\right)>1$. Specifically, in the public goods experiment, 1<γ<M, that is, 0<γ/M<1. Then, $\gamma -\left(\gamma /M\right)>1-\left(\gamma /M\right)$, which can be changed into the form of $M\cdot \left(\gamma /M\right)-\left(\gamma /M\right)>1-\left(\gamma /M\right)$. Then, we prove that $\left(M-1\right)\left(\gamma /M\right)/\left(1-\gamma /M\right)>1$, that is, δ>1. Therefore, the optimal contribution ratio of individual *i* is a linear function of the average contribution ratio of the other group members, which can be given by $x_{i}^{*}/w=\theta_{i1}\cdot \left(x_{e}/w\right)+\eta_{i1}$, where $\eta_{i1}=\left(\frac{a_{i}\cdot c}{a_{i}-b_{i}}/w\right)-\left[\frac{b_{i}}{a_{i}-b_{i}}/\left(1-\gamma /M\right)\right]$ and the slope is:

(6) $$\theta_{i1}=\frac{a_{i}-b_{i}\cdot \delta }{a_{i}-b_{i}}=1-\frac{\delta -1}{\left(a_{i}/b_{i}\right)-1}.$$

The parameter $a_{i}/b_{i}$ signifies an individual’s relative preference for material payoffs against his or her preference for complying with the legitimate behavior required by the conditional cooperation norm that is activated in the context. Moreover, the smaller the value of $a_{i}/b_{i}$, the stronger an individual’s relative respect for the legitimate behavior.

Regarding the slope of the optimal contribution ratio of individual *i* in the case of $0\leq x_{i}<x_{e}$, since δ>1, the slope $\theta_{i1}$ is smaller than 1 if $a_{i}/b_{i}>1$. This makes sense, as $a_{i}>b_{i}$ represents that individual *i* has a relatively weak preference for adhering to the legitimate behavior; thus, in this circumstance, individual *i* may display a negative deviation from the trend of perfect conditional cooperation when trading off monetary payoffs, which can be called imperfect conditional cooperation. Moreover, if $0<a_{i}/b_{i}<1$, then the slope of individual *i*’s contribution ratio $\theta_{i1}$ is larger than 1. As $a_{i}<b_{i}$ represents that individual *i* has a relatively strong preference for adhering to the legitimate behavior, the slope of his or her optimal contribution ratio is steeper than that of an individual with a weaker preference for adherence.

The second case is when $x_{e}\leq x_{i}\leq w$, that is, individual *i*’s contribution is not less than the legitimate behavior. Then, the utility function of *i* is as follows:

(7) $$u_{i}=a_{i}\ln\left[w-\left(1-\gamma /M\right)x_{i}+\left(\gamma /M\right)\left(M-1\right)x_{e}\right]+b_{i}\ln\left(c+x_{i}-x_{e}\right).$$

Similar to the analysis of the first case, we obtain a linear function of individual *i*’s optimal contribution ratio based on the other group members’ average contribution ratio, which is written by $x_{i}^{*}/w=\theta_{i2}\cdot \left(x_{e}/w\right)+\eta_{i2}$, where $\eta_{i2}=\left[\frac{b_{i}}{a_{i}+b_{i}}/\left(1-\gamma /M\right)\right]-\left(\frac{a_{i}\cdot c}{a_{i}+b_{i}}/w\right)$ and the slope is:

(8) $$\theta_{i2}=\frac{a_{i}+b_{i}\cdot \delta }{a_{i}+b_{i}}=1+\frac{\delta -1}{\left(a_{i}/b_{i}\right)+1}.$$

Since δ>1 and $a_{i}/b_{i}>0$, we verify that the slope of the optimal contribution ratio $\theta_{i2}$ is larger than 1 in the case of $x_{e}\leq x_{i}\leq w$. Furthermore, the smaller the value of $a_{i}/b_{i}$, the larger the value of $\theta_{i2}$, which demonstrates that individuals are more inclined to be conditionally cooperative if they have a relatively stronger preference for adhering to the legitimate behavior. Moreover, according to Equations (6) and (8), we find that only when the value of $a_{i}/b_{i}$ approaches infinity does the slope of individual *i*’s conditional contributions tend to be 1, in which case, perfect conditional cooperation is possible. Specifically, individuals who have internalized the norm of conditional cooperation generally do not match the others’ average contribution exactly and display a mild self-serving bias when trading off between material payoffs and adherence to the legitimate behavior, which is called imperfect conditional cooperation.

Based on the aforementioned analysis of the model, we find that, for an individual who has a preference for complying with the legitimate behavior required by the conditional cooperation norm, the optimal contribution rate is a linear function of the other group members’ average contribution rate. This, in turn, demonstrates that the behavioral pattern of the conditional cooperators in the public goods experiment designed by Fischbacher et al. [5] was derived from their preferences for complying with the legitimate behavior in that circumstance. In addition, according to Equations (6) and (8), we obtain a function of $a_{i}/b_{i}$, which represents an individual’s relative preference for complying with the legitimate behavior, as compared to his or her preference for material payoffs, on the slope $\theta_{i}$. Additionally, referring to the experimental design of Fischbacher et al. [5], we can observe each subject’s contribution choices given all the possible average contribution values of the other group members, which are integers within the interval of (0, 20), and calculate the slope of the 21 conditional contribution choices for each subject ($\theta_{i1}$ or $\theta_{i2}$). Subsequently, through Equation (6) or (8), we measure the extent of each subject’s respect for the legitimate behavior, relative to their preferences for material payoffs, and further discuss the distribution of these preferences among the subjects. In the following section, we describe our experimental design comprehensively.

### 3.2. Experimental Design and Procedures

Our experimental treatment was designed on the basis of a standard one-shot public goods experiment. Three participants were randomly matched as a group (M=3). Each subject had an initial endowment of 20 tokens and needed to decide the amount of tokens $x_{i}$, which was within the interval of (0, 20), to contribute to the group’s public account. The remaining endowment was kept as his or her private payoff. The total amount contributed to the group’s public account was multiplied by γ=1.5, and subsequently, equally shared by the three group members. Accordingly, the material payoff of individual *i* was written as:

(9) $$W\left(x_{i}\right)=20-x_{i}+0.5\sum_{j=1}^{3}x_{j}.$$

In our experimental treatment, the subjects were required to make two types of decisions sequentially, without knowing the decisions of the other group members. Firstly, they were requested to choose how many tokens to contribute to the group’s public account, which was called *unconditional contribution*. Secondly, we introduced the strategy method into the public goods experiment and instructed the subjects to complete a contribution table. Specifically, they needed to decide the amount that they were willing to contribute to the public account given all the possible average contribution levels of the other two group members, which were integers from 0 to 20. The second type of decision was called *conditional contribution*. To ensure that all the participants considered their decisions seriously, they were told that after all of them had made the two types of decisions, the experimental assistants would randomly select two of the three group members in each group for whom their unconditional contribution was relevant to the actual monetary payoffs calculation in the experiment; while for the other randomly selected member, his or her conditional contribution that corresponded to the average value of the other two members’ unconditional contributions was used for the actual payoffs calculation. Specifically, each group member was assigned a number between 1 and 3. For each group, the experimental assistants drew two sheets of paper from a box containing three sheets of folded paper with a number between 1 and 3 on them to determine the two group members whose unconditional contribution was payoff-relevant.

The experiment was conducted in a classroom at the Nanjing University of Science and Technology on 11 November 2019. Overall, 208 undergraduate students with different majors were recruited online. In fact, we recruited 210 undergraduates in total. However, two applicants decided not to participate in the experiment for private reasons at the last minute. Therefore, we invited other two subjects, who had participated in a similar experiment before, to participate in this experiment, which was unknown to other subjects. We did not incorporate the two subjects’ decisions into our analysis. The participants were required to read and approve the written informed consent before participating in the experiment. The duration of the experiment was approximately half an hour and the average payoff of the participants was RMB 21, including a show-up fee of RMB 5.

Upon arrival, the participants were handed an experimental instruction with a number on it and were subsequently seated in the position with the corresponding number. They were not allowed to talk to each other during the experiment. After all the participants were seated, the experimenter read the instructions aloud. Thenceforward, the participants first needed to take a quiz to make sure that they had fully understood the experiment. The questions were answered privately. Once all the participants had correctly completed the quiz, the experiment started. All the decisions were made on paper, and after all of them were finished, the experimental assistants collected the decision sheets. Subsequently, the participants were requested to complete a questionnaire. Meanwhile, the experimental assistants calculated each participant’s monetary payoff. The participants were paid privately in cash, post which, they exited the classroom.

## 4. Results

Our experiment aims at observing the subjects’ behavior in the conditional contribution situation, specifically, their contributions to the public good for each of the 21 possible values of the other group members’ average contributions. In this section, we firstly analyze the subjects’ behavioral patterns in the circumstance of conditional contribution and classify their behavioral patterns into different categories through the method introduced by Fischbacher et al. [5]; furthermore, we interpret the experimental results with our utility model. Subsequently, for the subjects who internalized the norm of conditional cooperation (conditional cooperators), we measure the parameter representing the degree of each subject’s respect for the legitimate behavior required by the norm, relative to their preference for monetary payoffs, and further discuss the distribution of the subjects’ heterogeneous preferences.

### 4.1. Identification of the Behavioral Patterns in the Conditional Contribution Situation

**Figure 1:**
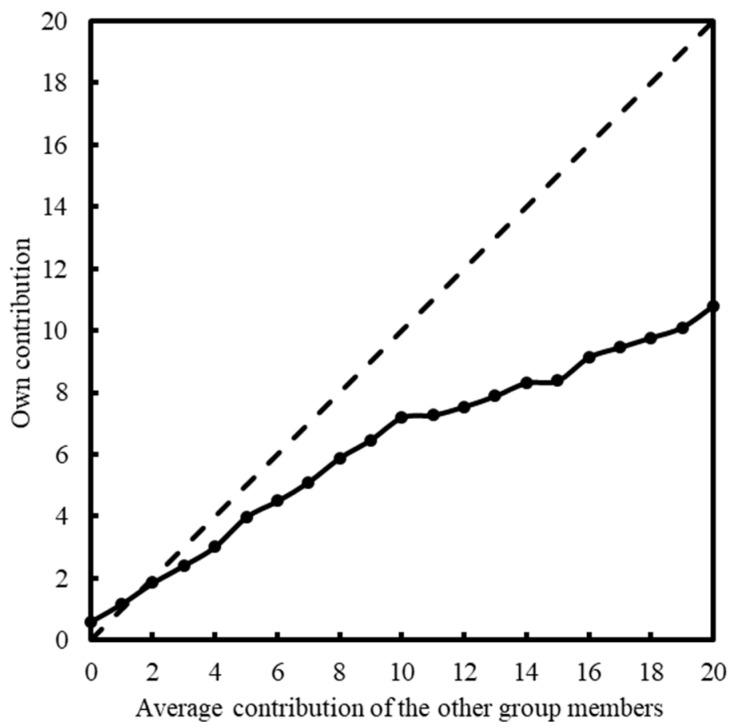
Total average of own contribution for each average contribution level of the other group members.

We primarily consider the subjects’ average contribution levels under each of the 21 conditions. As shown in Figure 1, the dashed line represents the behavioral pattern of perfect conditional cooperation; the total average contribution level of the subjects increases with the given value of the other group members’ average contribution, however, it negatively deviates from the perfect conditional cooperation’s trend. Thus, overall, the subjects display an obvious propensity to cooperate conditionally.

However, the individual-level data show heterogeneity in the subjects’ conditional contribution patterns. Therefore, referring to the classical classification criteria [5], we divide the subjects’ behavioral patterns into four categories according to their contributions under the 21 conditions, which are free-riding, conditional cooperation, hump-shaped patterns, and other patterns. The results are displayed in Table 1. In our experiment, the behavioral patterns of 34 subjects (16.3%) fall into the category of free-riding, where they contribute nothing under all the conditions. Furthermore, 121 subjects (58.2%) are conditionally cooperative, where their contribution is positively correlated with the mean value of the other group members’ contributions, with the Spearman rank correlation coefficient being significant at the 0.001 level. The behavioral patterns of 19 subjects (9.2%) are hump-shaped, where, although they first increase their contribution with the mean value of the others’ contributions, they reduce it after the others’ mean contribution is beyond a certain level. The behavioral patterns of the other 34 subjects (16.3%) cannot be classified into the above-mentioned three categories. As shown in Table 1, the distribution of the subjects’ behavioral patterns in our experiment is similar to that in Fischbacher et al.’s [5] research, indicating that our experimental implementation and results are reasonable. Besides, Fischbacher and Gächter [4] increased the number of subjects and found that the proportion of the conditional cooperators was 55%, which was similar to Fischbacher et al. [5]. Rustagi et al. [6] provided field evidence among actual commons users and demonstrated the consistency of people’s propensities for conditional cooperation in the laboratory and in reality. Vollan et al. [37] also found that this type of conditional cooperation is stable among different groups of people in China. In their experiment, the proportion of the conditional cooperators was about 50% in a sample of both students and workers. The distribution of the conditional cooperation in this paper is similar to that in these previous studies, which confirms the rationality of our experimental implementation and results. We also measured the subjects’ unconditional contributions in the experiment. The average contribution of the conditional cooperators (7.35) was significantly higher than that of the free-riders (4.50), with *p* < 0.01. The average contributions of the hump-shaped subjects and other subjects were 6.11 and 7.03, respectively.

Among the four categories, the behavioral patterns of the free-riders and conditional cooperators are linear and can be interpreted by our utility model. Specifically, the behavior of about 75% of the subjects in our experiment is consistent with the predictions of the model. According to the analysis of our log-linear utility function, purely selfish individuals are insensitive to deviations from the legitimate behavior and only care about their own monetary payoffs. Therefore, regardless of how much the others contribute to the public good, the optimal choice of these individuals contributes nothing. In our experiment, 16.3% of the subjects who display the behavioral pattern of free-riding are purely self-interested. Moreover, more than half of the subjects (58.2%) are conditionally cooperative, where their contribution level has a significant linear correlation with the average of the others’ contributions. As assumed by our model, individuals who have internalized this conditional cooperation norm regard the other group members’ average contributions as the legitimate behavior they should take, which is required by the norm activated in the conditional contribution situation. Deviating from the legitimate behavior may cause a utility loss for such people. Additionally, they will trade-off between monetary payoffs and adherence to the legitimate behavior when making their choices. Accordingly, we obtain from the model that, for individuals who have a preference for complying with the legitimate behavior that corresponds to the conditional cooperation norm, their optimal contribution ratio is a linear function of the others’ average contribution ratio. That is, the behavioral pattern of the conditional cooperation prevalent in the experiment can be explained by the utility function constructed in this paper, which contains the legitimate behavior and its deviation. Furthermore, regarding the conditional cooperators, we then measure the extent of each subject’s respect for the legitimate behavior, relative to their preference for monetary payoffs, through the utility function and their conditional contributions.

### 4.2. Measurement of Participants’ Relative Respect for the Legitimate Behavior

Among the 121 conditional cooperators, 27 subjects are perfectly conditionally cooperative, meaning they contribute exactly the same as the others’ average contribution, accounting for 22% of the conditional cooperators. In the experiment by Fischbacher et al. [5], 4 of the 22 conditional cooperators were perfectly conditionally cooperative, accounting for 18.2%. Our results are similar with theirs, which is reasonable. According to the analysis of the utility function in Section 3.1.2, we learned that individuals who are sensitive to deviations from the legitimate behavior generally display “imperfect conditional cooperation” when trading off between material payoffs and their compliance with the legitimate behavior. Only when the value of $a_{i}/b_{i}$ approaches infinity will the slope of an individual’s conditional contributions tend to be 1, that is, perfectly conditional cooperation. Therefore, the perfect conditional cooperators’ relative degrees of respect for legitimacy cannot be measured through the experimental data in this paper. Thus, in the rest of this section, we only analyze the conditional contribution data of the other 94 conditional cooperators.

Based on the equilibrium analysis of the utility function in Section 3.1.2, the value of the parameter $a_{i}/b_{i}$, which represents an individual’s relative respect for the legitimate behavior, is calculated according to the following three conditions.

Condition I. If the actual contribution ratio of the subjects is not lower than the average contribution ratio of the other group members under all 21 given conditions, that is, $x_{i}/w\geq x_{e}/w$, the parameter of this type of subjects is calculated through Equation (8), written by $a_{i}/b_{i}=\left(\delta -1\right)/\left(\theta_{i}-1\right)-1$, where $\theta_{i}>1$. In our experimental setting, the value of δ is equal to 2.

Condition II. If the actual contribution ratio of the subjects is lower than the average contribution ratio of the other group members under all 21 given conditions, that is, $x_{i}/w<x_{e}/w$, the parameter of this type of subjects is calculated through Equation (6), written as $a_{i}/b_{i}=1+\left(\delta -1\right)/\left(1-\theta_{i}\right)$.

Here, if $\theta_{i}<1$, then the parameter is written as $a_{i}/b_{i}=1+\left(\delta -1\right)/\left(1-\theta_{i}\right)$, which is larger than 1; if $\theta_{i}>1$, then the parameter can be changed into the form of $a_{i}/b_{i}=1-\left(\delta -1\right)/\left(\theta_{i}-1\right)$, which is smaller than 1.

Condition III. If the actual contribution ratio of the subjects has both the case of being greater than or equal to the others’ average contribution ratio and the case of being lower than the others’ average contribution ratio, the parameter of this type of subjects is calculated as follows:

if $\theta_{i}<1$, $a_{i}/b_{i}=1+\left(\delta -1\right)/\left(1-\theta_{i}\right)$;
if $\theta_{i}>1$, $a_{i}/b_{i}=\left|\left(\delta -1\right)/\left(\theta_{i}-1\right)-1\right|$.

Hence, we first calculate the slope of each subject’s 21 conditional contributions. Subsequently, we divide the 94 conditional cooperators into three categories according to the aforementioned three conditions and measure their relative respect for the legitimate behavior separately. The detailed results are as follows.

**Figure 2:**
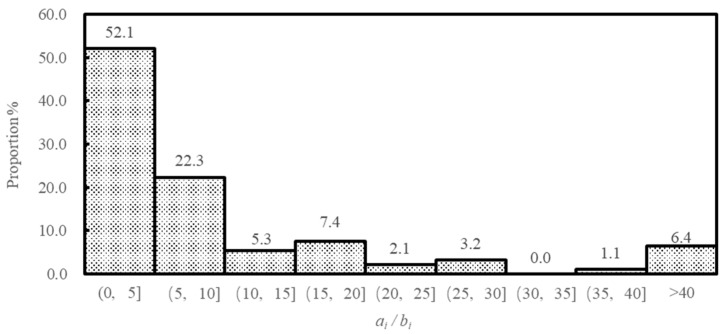
Distribution of the value of $a_{i}/b_{i}$. $a_{i}/b_{i}$ represents individual *i*’s relative degree of respect for the legitimate behavior. The smaller the value of $a_{i}/b_{i}$, the higher the relative degree of one’s respect for the legitimate behavior.

In our experiment, there are 7 subjects whose conditional contributions satisfy Condition I, that is, they do not contribute less than the average amount of the others’ contributions in all cases. The measurement results indicate that the slopes of these subjects’ conditional contribution ratios are all greater than 1. Regarding the corresponding value of the parameter $a_{i}/b_{i}$, the mean value of these subjects is 13.5, with the maximum and minimum values being equal to 29.8 and 3.7, respectively. Furthermore, among these subjects, the larger the value of $a_{i}/b_{i}$, the smaller the value of $\theta_{i}$. Additionally, the conditional contributions of the remaining 87 conditional cooperators all satisfy Condition III; the mean value of the parameter $a_{i}/b_{i}$ of these subjects is 16.6. Among these subjects, there are 69 subjects whose slope of conditional contributions is smaller than 1, with the mean value of the parameter $a_{i}/b_{i}$ being equal to 15.0; moreover, there are 18 subjects whose slope of conditional contributions is larger than 1, with the mean value of $a_{i}/b_{i}$ being equal to 22.6. Furthermore, the distribution of the $a_{i}/b_{i}$ parameter’s values for the 94 conditional cooperators, that is, the distribution of their relative degrees of adherence to the legitimate behavior required by the conditional cooperation norm, is displayed in Figure 2. The smaller the value of $a_{i}/b_{i}$, the higher the relative degree of one’s respect for the legitimate behavior. We find that these subjects’ relative degrees of adherence are highly centrally distributed. Specifically, the values of $a_{i}/b_{i}$ for 74.4% of the subjects are concentrated in the interval of (0, 10); that is, most subjects have a relatively high degree of adherence to the legitimate behavior, as compared to their preference for monetary payoffs. Additionally, a small fraction of the subjects (6.4%) have a relatively low sensitivity to deviations from the legitimate behavior, with the value of their parameter $a_{i}/b_{i}$ being above 40.

## 5. Discussion

Formal frameworks based on social preference theories mainly assume that the utility of a decision maker is a function of the material payoff caused by the available choices. However, many empirical results (such as the framing effect) cannot be entirely explained by utility theories based solely on people’s preferences for the relative distribution of material payoffs. To solve this issue, we propose that people have preferences for following the behavioral norm embedded in a given context. Furthermore, this norm implies what the right thing to do is in a given context, which this study refers to as the legitimate behavior. Specifically, we assume that people have preferences for complying with this legitimate behavior beyond the material payoffs caused by their behavior; moreover, they do so in a context-sensitive manner. In our model, individuals may suffer a utility loss due to experiencing unpleasant emotions such as guilt or remorse if they negatively deviate from the legitimate behavior; they will trade-off between monetary payoffs and adherence to the legitimate behavior when making their choices. Furthermore, the sensitivity to these deviations is heterogeneous across individuals. Those who are more sensitive to such deviations will make choices more consistent with the legitimate behavior. Based on our model, the puzzling empirical results can be interpreted as the heterogeneity in the legitimate behavior or the behavioral norm activated by different context signals.

Subsequently, we apply our model to the case of conditional contributions in a one-shot public goods experiment so that we can formalize the preference of conditional cooperation and further understand the micro mechanism underlying the behavioral patterns of conditional cooperation. The equilibrium analysis of the utility function indicates that, for individuals who have internalized the norm of conditional cooperation, their optimal choice is a linear function of the others’ average contribution. This result in turn validates that the behavioral pattern of conditional cooperation in a public goods experiment is derived from individuals’ preferences for adhering to the legitimate behavior, which corresponds to the conditional cooperation norm activated by the experimental context.

In addition, based on the analysis of the utility function, we obtain an equation of the parameter that represents an individual’s preference for adhering to the legitimate behavior, relative to his or her preference for monetary payoffs, in the context of conditional contributions. In order to provide a more detailed picture of the distribution of individuals’ heterogeneous degrees of adherence to a legitimate behavior, we conducted an experiment similar to the conditional contribution experiment by Fischbacher et al. [5] and utilized the observable conditional contribution data to measure the parameter that represented an individual’s relative sensitivity to deviations from the legitimate behavior, for each subject among the conditional cooperators. The measurement results revealed that the subjects’ relative sensitivities to these deviations are highly centrally distributed; furthermore, most subjects have a relatively high degree of adherence to the legitimate behavior in the experimental context of conditional contribution.

Overall, this study helps in understanding individuals’ behavior by introducing legitimate behavior and its deviation into a formal utility function. Based on the model, we can further comprehend the micro mechanism underlying conditional cooperation. Additionally, we attempted to measure the individual-level respect for the legitimate behavior using the observable experimental data, since the previous related literature has focused either on estimating the population’s average concern for behavioral norms or on measuring individuals’ preferences for the relative distribution of material payoffs caused by the behavior.

Moreover, there are two other issues worthy of future exploration. First, this research measures the individual-level respect for the legitimate behavior in the conditional contribution context of a public goods experiment; however, we have not verified whether the measurement results can account for the subjects’ behavior in other decision situations, that is, whether the degree of an individual’s respect for the legitimate behavior is stable across different contexts. This remains to be validated by subsequent research. Second, in the case of conditional contributions in the public goods experiment, we construct our utility function by assuming the legitimate behavior taken by individuals who have internalized the norm embedded in the context; in the following studies, we should attempt to measure individuals’ evaluations on the legitimacy of the available choices in a given context, which will help to confirm our assumptions and improve the integrity of the research.

## Figures and Tables

**Table 1:** Distribution of the behavioral pattern of conditional contributions.

Behavioral Pattern	This Paper		Fischbacher et al. (2001)	
	N	Proportion (%)	N	Proportion (%)
Free-riding	34	16.3	13	29.6
Conditional cooperation	121	58.2	22	50
Hump-shaped patterns	19	9.2	6	13.6
Other patterns	34	16.3	3	6.8
Total	208	100	44	100

## Data Availability

The data that support the findings of this study are available on request from the corresponding author.

## References

[1] Fehr E., Fischbacher U. (2004). Social norms and human cooperation. Trends Cogn. Sci..

[2] Elster J. (1989). Social norms and economic theory. J. Econ. Perspect..

[3] Camerer C.F., Fehr E., Henrich J., Boyd R., Bowles S., Camerer C., Fehr E., Gintis H. (2004). Measuring social norms and preferences using experimental games: A guide for social scientists. Foundations of Human Sociality: Economic Experiments and Ethnographic Evidence from Fifteen Small-Scale Societies.

[4] Fischbacher U., Gächter S. (2010). Social preferences, beliefs, and the dynamics of free riding in public goods experiments. Am. Econ. Rev..

[5] Fischbacher U., Gächter S., Fehr E. (2001). Are people conditionally cooperative? Evidence from a public goods experiment. Econ. Lett..

[6] Rustagi D., Engel S., Kosfeld M. (2010). Conditional cooperation and costly monitoring explain success in forest commons management. Science.

[7] Thöni C., Tyran J.R., Wengström E. (2012). Microfoundations of social capital. J. Public Econ..

[8] Fehr E., Schmidt K.M. (1999). A theory of fairness, competition, and cooperation. Q. J. Econ..

[9] Edgeworth F.Y. (1881). Mathematical Psychics: An Essay on the Application of Mathematics to the Moral Sciences.

[10] Rabin M. (1993). Incorporating fairness into game theory and economics. Am. Econ. Rev..

[11] Charness G., Rabin M. (2002). Understanding social preferences with simple tests. Q. J. Econ..

[12] List J.A. (2007). On the interpretation of giving in dictator games. J. Political Econ..

[13] Falk A., Fehr E., Fischbacher U. (2003). On the nature of fair behavior. Econ. Inq..

[14] Krupka E.L., Weber R.A. (2013). Identifying social norms using coordination games: Why does dictator game sharing vary?. J. Eur. Econ. Assoc..

[15] Capraro V., Vanzo A. (2019). The power of moral words: Loaded language generates framing effects in the extreme dictator game. Judgm. Decis. Mak..

[16] Tversky A., Kahneman D. (1981). The framing of decisions and the psychology of choice. Science.

[17] Tversky A., Kahneman D. (1992). Advances in prospect theory: Cumulative representations of uncertainty. J. Risk Uncertain..

[18] Lapinski M.K., Rimal R.N. (2005). An explication of social norms. Commun. Theory.

[19] Capraro V., Perc M. (2021). Mathematical foundations of moral preferences. J. R. Soc. Interface.

[20] Kimbrough E.O., Vostroknutov A. (2016). Norms make preferences social. J. Eur. Econ. Assoc..

[21] Kimbrough E.O., Vostroknutov A. (2018). A portable method of eliciting respect for social norms. Econ. Lett..

[22] Dufwenberg M., Kirchsteiger G. (2004). A theory of sequential reciprocity. Games Econ. Behav..

[23] Falk A., Fischbacher U. (2006). A theory of reciprocity. Games Econ. Behav..

[24] Bolton G.E., Ockenfels A. (2000). ERC: A theory of equity, reciprocity, and competition. Am. Econ. Rev..

[25] Andreoni J. (1995). Warm-glow versus cold-prickle: The effects of positive and negative framing on cooperation in experiments. Q. J. Econ..

[26] Smith A. (1759). The Theory of Moral Sentiments.

[27] Zhang W., Zhu X., Guan H., Li T. (2020). Self-Conscious or fear of hurting another’s feeling? An experimental investigation on promise-keeping. Front. Psychol..

[28] Levitt S.D., List J.A. (2007). What do laboratory experiments measuring social preferences reveal about the real world?. J. Econ. Perspect..

[29] López-Pérez R. (2008). Aversion to norm-breaking: A model. Games Econ. Behav..

[30] Kessler J.B., Leider S. (2012). Norms and contracting. Manag. Sci..

[31] Capraro V., Jagfeld G., Klein R., Mul M., van de Pol I. (2019). Increasing altruistic and cooperative behaviour with simple moral nudges. Sci. Rep..

[32] Bicchieri C., Chavez A. (2010). Behaving as expected: Public information and fairness norms. J. Behav. Decis. Mak..

[33] Tyler T.R. (2006). Why People Obey the Law.

[34] Andrighetto G., Grieco D., Tummolini L. (2015). Perceived legitimacy of normative expectations motivates compliance with social norms when nobody is watching. Front. Psychol..

[35] Murphy R.O., Ackermann K.A., Handgraaf M. (2011). Measuring social value orientation. Judgm. Decis. Mak..

[36] Ledyard J.O., Kagel J., Roth A. (1995). Public Goods: A Survey of Experimental Research. Handbook of Experimental Economics.

[37] Vollan B., Landmann A., Zhou Y., Hu B., Herrmann-Pillath C. (2017). Cooperation and authoritarian values: An experimental study in China. Eur. Econ. Rev..

